# Improving the efficiency of phosphate rocks combined with phosphate solubilizing Actinomycetota to increase wheat growth under alkaline and acidic soils

**DOI:** 10.3389/fpls.2023.1154372

**Published:** 2023-05-10

**Authors:** Kenza Boubekri, Abdoulaye Soumare, Karim Lyamlouli, Yedir Ouhdouch, Mohamed Hafidi, Lamfeddal Kouisni

**Affiliations:** ^1^ AgroBioSciences Department (AgBS), Mohammed VI Polytechnic University (UM6P), Benguerir, Morocco; ^2^ Laboratory of Microbial Biotechnologies Agrosciences and Environment (BioMAgE), Labelled Unit CNRST N°4, Faculty of Sciences Semlalia, Cadi Ayyad University (UCA), Marrakech, Morocco; ^3^ Laboratory of Agroforestry and Ecology, Assane Seck University (UASZ-UFR ST), Ziguinchor, Senegal; ^4^ African Sustainable Agriculture Research Institute (ASARI), Mohammed VI Polytechnic University (UM6P), Laayoune, Morocco

**Keywords:** Actinomycetota, rock phosphate, wheat plant growth, acid and alkaline soil, nutrient uptake

## Abstract

Low availability of phosphorus (P) in both acidic and alkaline soils is a major problem for sustainable improvement in wheat crops yield. Optimization of crops productivity can be achieved by increasing the bioavailability of P by phosphate solubilizing *Actinomycetota* (PSA). However, their effectiveness may vary with changing agro-climatic conditions. In this regard, a greenhouse experiment was conducted to assess the interaction inoculation of five potential PSA (P16-P18-BC3-BC10 and BC11) and RPs (RP1- RP2-RP3 and RP4) on the growth and yield of wheat crop in unsterilized P- deficient alkaline and acidic soils. Their performance was compared with single super phosphate (TSP) and reactive RP (BG4). The *in-vitro* tests showed that all PSA colonize wheat root and form a strong biofilm except *Streptomyces anulatus* strain P16. Our findings revealed that all PSA significantly improve the shoot/root dry weights, spike biomass, chlorophyll contents as well as nutrients uptake in plants fertilized with RP3 and RP4. However, the combined application of *Nocardiopsis alba* BC11 along with RP4 in alkaline soil, was effective in optimizing wheat yield attributes and improve the yield biomass up to 19.7% as compared to the triple superphosphate (TSP). This study supports the view that the inoculation with *Nocardiopsis alba* BC11 has a broad RP solubilization and could alleviate the agricultural losses due to P limitation in acidic and alkaline soils.

## 1 Introduction

The bioavailability of major plant nutrients, especially phosphorus (P), affects plant growth and yield (Fageria and Nascente, 2014). Several reports have shown that the deficiency of P has become a threat to soil fertility and crop productivity affecting 30-50% of the cultivated land in the world and causing a yield loss in the range of 10% to 15% (Shenoy and Kalagudi, 2005; Ringeval et al., 2017). The direct application of RP, as an alternative P source, is currently attracting increased interest due to its relatively low costs and its utilization potential (Vuuren et al., 2010; Hellal et al., 2019). Nonetheless, its low solubility is a major obstacle to its direct application, especially in alkaline soils (Arcand and Schneider, 2006). Therefore, developing novel strategies to enhance RP solubilization and improve its agronomic efficiency has become a pivotal research challenge (Veneklaas et al., 2012; Numan et al., 2018; Yagi et al., 2020). A considerable number of soil microorganisms from bacterial genera (*Bacillus*, *Pseudomonas*, and *Rhizobium*) and fungal genera (*Penicillium* and *Aspergillus*) are effective in releasing P from total soil phosphorus through solubilization/mineralization (Kalayu, 2019; Fahsi et al., 2021; Mahdi et al., 2021a; Mahdi et al., 2021b). These phosphate solubilizing microorganisms (PSM) are believed to provide an eco-friendly and economically sound approach to overcome the P scarcity (Pathak et al., 2017; Anand et al., 2023). PSM also play a dominant role in the plant growth *via* the synthesis and through a secretion of a plethora of beneficial substances such as auxins, cytokinins, and gibberellic acid, as well as ethylene, hydrogen cyanide, and siderophores (Wahid et al., 2020; Yu et al., 2022). These secondary metabolites are well documented to precisely match the plant’s needs and safeguard plants from pathogen’s infection (Yu et al., 2020; Chaudhry et al., 2021; Mowafy et al., 2022). Application of such naturally occurring organisms possessing multiple growth-promoting activities holds therefore greater promise for increasing the productivity of many crops (Wang et al., 2022). Among plant-growth promoting bacteria, *Actinomycetota* have been reported to increase P solubilization in soil by decreasing the soil pH through the production of organic acids, phytohormones, chelating agents and siderophores (Hamdali et al., 2008; Soumare et al., 2020a; Soumare et al., 2020b; Boubekri et al., 2021). With their abilities to produce spores and to survive in very competitive environments, *Actinomycetota* are considered the most advantageous and suitable candidates for the production of highly versatile biofertilizers (Boubekri et al., 2022). Furthermore, these filamentous microorganisms are known for improving plant tolerance to biotic and abiotic stresses and enhancing nutrient availability and uptake (Bhatti et al., 2017; El-Badan et al., 2019; El-Tarabily et al., 2020). However, the performance of these plant growth promoting bacteria is severely influenced by environmental factors such as soil pH. The composition and functionalities of the microbial population are affected under soil alkaline or acidic conditions, which induces changes in the nutrient dynamic (Nicol et al., 2008; Souza et al., 2015; Neina, 2019). In this regard, variation of soil pH is considered not only the main driving force for plant growth but also an important biomarker for P availability. The available forms of P for plants (H_2_PO_4_^-^ and HPO_4_^2-^) are maximized at two main pH conditions: pH 4.5 and 6.5, where the degree of P fixation by calcium (Ca), aluminum (Al), and iron (Fe) is minimized (Penn and Camberato, 2019; Bouray et al., 2021). *Actinomycetota* can extend the broader P solubilization spectrum. Interestingly, our previous findings had already shown that *Actinomycetota* inoculations not only improved wheat/maize crops but also improved significantly the NPK statue of the plants (Soumare et al., 2020a; Boubekri et al., 2021). However, their combined use in releasing P from RP in unsterilized alkaline and acidic soils have been little investigated. Therefore, it is challenging to explore the effects of different application of RPs fertilized with *Actinomycetota* strains on growth and yield of wheat crops in a complex environmental condition using natural (unsterilized) alkaline and acidic soils. In this study, we hypothesized that combined use of *Actinomycetota* with RP is better approach to improve wheat growth and yield and could be an efficient biofertilizer adaptable for different soil types. Therefore, the main objective of this study was to evaluate the effect of *Actinomycetota*-RP-soil pH combinations on wheat plant growth in non-sterile soil’s conditions. Overall, the specific objectives of this study are as follows:

- i. Evaluate the effect of five Actinomycetota strains on the solubilization of four RPs grades in natural soil condition.
- ii. Investigate the effect of soil pH on the stimulatory effect of Actinomycetota-RPs combinations to promote wheat plant growth under greenhouse conditions.
- iii. Assess the effect of the Actinomycetota-RPs combinations on nutrients uptake acquisitions.
- iv. Suggest an environment-friendly P fertilizer based on *Actinomycetota* and RP adapted for P-deficient alkaline and acidic soils.

The findings of this study could provide an effective approach for agronomic improvement of *Actinomycetota* inoculants to enhance RP solubilization and promote wheat plant growth, either in acidic or alkaline soils.

## 2 Materials and methods

### 2.1 PGPR characteristics of the microbial strains

Strains used in this study were obtained from the Laboratory of Biotechnology, Faculty of Science Cadi Ayyad of Marrakech. Bacterial strains *S. anulatus* (P16), *S. alboviridis* (P18, BC3), *S.griseorubens* (BC10) and *N.alba* (BC11) were isolated from desert soil of Morocco and were previously selected for their ability to solubilize different grade of RPs (RP1, RP2, RP3 and RP4) and to stimulate plant growth in *in-vitro* (**Table 1**) (Soumare et al., 2020a; Boubekri et al., 2021).

**Table 1:** Plant Growth promoting Traits of the *Actinomycetota* strains.

Strains	Max RP solubilization (mg/l)				Potassium solubilsation (mg/l)	Max AIA production	Siderophore production	HCN	Ammonia	Accession number
	RP1	RP2	RP3	RP4						
**P16**	1.6 ± 0.2a	5.9 ± 0.34b	3.1 ± 0.22d	14.1 ± 0.78c	–	57.73± 0.89c	1.555 ± 0.00b	++	+	MT845227
**P18**	0.7 ± 0.1cd	11.5 ± 0.82a	30.9 ± 0.05a	31.5 ± 0.35a	3 ± 0.07d	82.92 ± 1.5b	1.477 ± 0.28ab	++	++	MT845229
**BC3**	0.8 ± 0.05c	7.2 ± 0.54ab	30.9 ± 0.13a	30.1 ± 0.33ab	11 ± 0.52c	10.02 ± 2.34d	1.736 ± 0.00a	+	+	MT845230
**BC10**	0.3 ± 0.17d	5.9 ± 0.76b	26.7 ± 0.6b	31.2 ± 0.17a	12.73 ± 0.35b	128.44 ± 4.08a	1.348 ± 0.00c	++	++	MT845231
**BC11**	1.2 ± 0.1b	6.7 ± 0.83ab	21.7 ± 1.2c	7.7 ± 0.54d	17.8 ± 1.02a	82.33 ± 0.84b	1.552 ± 0.00b	+	+	MT845232

RP(1, 2, 3 and 4) composition see (Boubekri et al., 2021). Different letters indicate significant differences (p <0.05).

+, high production; ++, very high production.

### 2.2 Root colonization potential of *Actinomycetota* strains

The ability of the selected *Actinomycetota* strains (P16 -P18 -BC3 -BC10 and BC11) to colonize wheat seed teguments was assessed using scanning electron microscopy. The wheat seeds (*Triticum aestivum* variety *Vitron)* were surface sterilized with 1% sodium hypochlorite for 1 min and washed several times with sterile distilled water. The sterilized seeds were germinated in the dark for 48h on Petri dish containing agar gel (0.7%). The germinated seeds were treated with the *Actinomycetota* inoculums (P16, P18, BC3, BC10 and BC11; at 10^8^ CFU ml^-1^) for 12h, sown in the pots containing sterilized coarse sand, and incubated in a growth chamber for 15 days (Bringel, 1997; Miranda, 1997). At the end of the incubation, wheat seedlings were removed carefully from the pots and the roots were washed in 0.1 M phosphate buffer (pH 7.2). The tip of the roots was cut into 4-5 mm long pieces and fixed in 2.5% glutaraldehyde, 0.1M phosphate buffer (pH 7.2) for 24h at 4°C. Thereafter, the samples were dehydrated using a graded series of ethanol solutions (30-100%). The dehydrated samples were then freeze dried to avoid desiccation following the protocol of (Gopalakrishnan et al., 2015). The processed samples were mounted and coated with a thin layer of gold using an automated sputter coater for 5 min and further scanned using the scanning electron microscopy (SEM) Zeiss EVO 10 (Carl Zeiss Microscopy, GmbH, Jena, Germany). The samples were operated at an accelerating voltage of 10/20.00 kV.

### 2.3 Biofilm production assay

Biofilm formation was assessed using the colorimetric assay (Christensen et al., 1985). Fresh overnight culture of each *Actinomycetota* strains was diluted in tryptic soy broth (TSB) and 200 µl of each bacterial suspension (OD= 1) was inoculated in triplicate into a 48-well microtiter microplate. Uninoculated media was used as negative control and *Pseudomonas aeruginosa* suspension as a positive control. The microplate was incubated at 38°C for 24h. The supernatants were aspirated using VACUSIP system and the bacterial pellets were washed three times with 200 µl of phosphate-buffered saline (PSB). Afterwards, 2% of crystal violet was added to each well for 20-40 min at room temperature to monitor the biofilm formation. The excess dye was washed out with distilled water. The bacterial biofilm was solubilized using 200 µl of 95% ethanol and the OD_600nm_ was measured using the VICTOR Nino ™ Multimode Plate Reader. The OD values were taken as an index of biofilm formation. The OD_c_ of the control (uninoculated media) was subtracted from the OD_T_ obtained in each treatment.

### 2.4 Greenhouse experiment design

A pot experiment was carried out to investigate the effects of *Actinomycetota*-RPs combinations on wheat growth in acidic and alkaline soils. Four different RPs (RP1, RP2, RP3 and RP4) containing between 27.46% and 32.81% of P_2_O_5_ were used in this study. The RPs were sieved (diameter between 100 and 200 µm) and washed to remove the P available fractions. The experiment was conducted from January 2019 to April 2020 at the experimental farm of the Mohammed VI Polytechnic University, Benguerir, Morocco. The acidic soil (sandy) was collected in the experimental field of the National Institute of Agronomic Research (INRA) in Laarache region, Morocco, while the alkaline one (clay-loam) was taken from Marrakech region, Morocco. Their chemical properties are presented in **Table 2**.

**Table 2:** Chemical properties of alkaline and acidic soils.

Soil types	pH	pH _KCL_	EC (mS/cm)	Total Nitrogen (%)	C _org_ (%)	P (mg/kg)	K_2_O(mg/kg)
**Acidic**	5.8	5.57	0.03	0.04	0.83	8	168
**Alkaline**	9	ND	0.19	ND	1	11	ND

ND, Not determined.

Six sterilized wheat seeds were sown in plastic pots, each pot was previously filled with 4.5 Kg unsterilized soils. After germination, plants were thinned to four per pot. The pots were arranged in a completely randomized block design (RCBD) with 27 treatments and 5 replications. For each type of soil, controls and inoculations treatments were carried out. The control treatments are distributed as follows: **(1)** (C^-^) negative control (without bacterial inoculation nor RP fertilization); **(2)** C^+^ (TSP) positive control containing triple superphosphate (containing 46% of soluble P_2_O_5_) and (3) BG4 reactive rock phosphate (29.75% of P_2_O_5_) as a second positive control. The inoculated treatments consist of a combination of strain (*S. anulatus* noted P16, *S. alboviridis* noted P18, *S. griseorubens* noted BC3, *S. griseorubens* noted BC10 and *N. alba* noted BC11) and RP (RP1, RP2, RP3 and RP4). Microbial inoculation was performed after 7 days of emergence by adding 2 mL of each *Actinomycetota* suspension (OD = 1 corresponding to 7. 10^8^ CFU) in the seedling rhizosphere vicinity. The pots were watered regularly to maintain the soil at field capacity. The TSP fertilizer was applied at the recommended rate of 130 kg/ha which provides 60 kg P_2_O_5_/ha. The amount of RP providing the same amount of P_2_O_5_ was determined by considering the total P content of each RP. To complete the essential needs of the crop, nitrogen (N) and potassium (K) were brought in the form of fertilizers with the respective doses of 100 Kg/ha for N, and 80 Kg/ha for K.

The percentage increment (IC) of shoot, root, and spike of the *Actinomycetota*-RP inoculation was calculated according to the following formula:


. $$\%\text{IC}=\frac{\text{Y}\left(Actinomycetota-\text{RP combination}\right)-\text{Y}\left(\text{BG}4\text{l}\right)}{\text{Y }\left(\text{BG}4\right)}\times 100$$

Where Y (*Actinomycetota*-RP combination) is biomass yield from the application of the *Actinomycetota*-RP combination and Y(BG4) is biomass yield from the positive control BG4.

### 2.5 Plant analysis

After 4 months, the plants have been removed and adhering particles were washed with distilled water. Shoot, root dry weights and spike biomass were measured after drying in a forced-air oven at 72°C for 48h. Thereafter, the dry leaves were finely ground and homogenized to determine the P and K concentrations. Each sample (0.5 g) was digested and analyzed for P content according to the Molybdo-phosphoric blue method (Murphy and Riley, 1962). P uptake per pot was calculated by multiplying biomass (g) by P concentrations (mg/g). The residual phosphorus in the soil was determined at harvest according to Olsen (1954) method. The available K was determined by atomic absorption spectrometer (SAA). The chlorophyll content was measured from the middle part of the leaf using CL-O1 chlorophyll meter (Hansatech instruments). For every measurement, the same part of the leaf was placed between two clips and the chlorophyll content index was determined in dual wavelength optical absorbance (620 and 940 nm).

### 2.6 Statistical analysis

The data were collected in five replicates and subjected to one-way ANOVA to examine the significance of differences and variability at 95% confidence level (*p<0.05*). The Pearson correlations between the plant growth parameters were determined using SPSS 22. Software. Multivariate analyses were applied to obtain more insight into the data matrix. Principal component analysis (PCA) was performed to examine how combined soil and rock phosphates influenced the biological attributes of the *Actinomycetota* strains, and to determine which inter-related parameters that influenced more the plant growth promoting (PGP) potential of the strains. The PCA, boxplots, and the effect size analysis were performed using R statistical package 3.2.5 (R Foundation for Statistical Computing). The graphics were performed using GraphPad Prism 8 software.

## 3 Result

### 3.1 Root colonization and biofilm production of *Actinomycetota* inoculums

**Figure 1:**
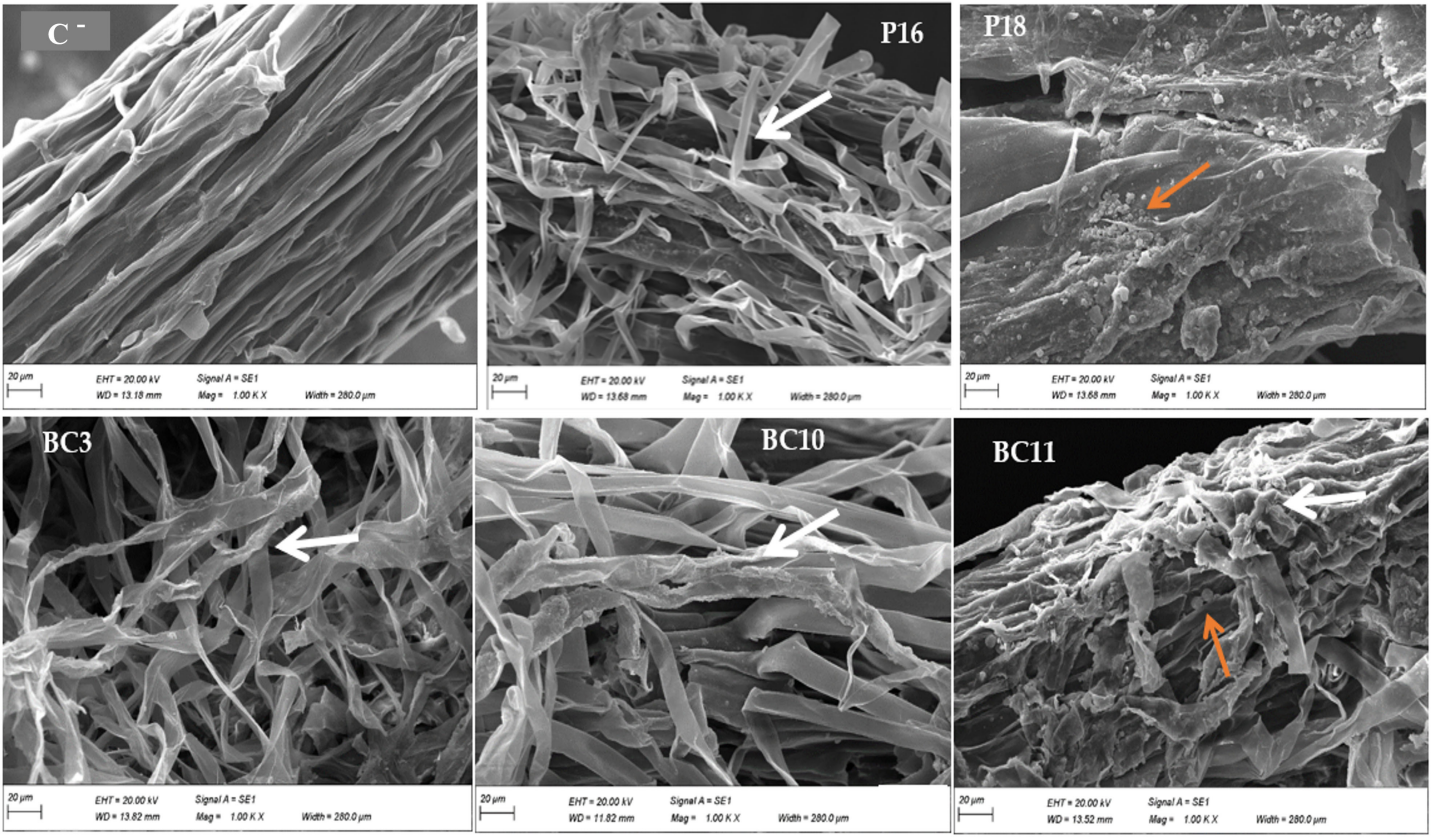
Root coloniztion by the *Actinomycetota* strains (P16, P18, BC3, BC10 and BC11) after 15 days of inoculations by scanning electron microsopy. Non-bacterized (C^-^) root is shown in a. Insets show *Actinomycetota* attached to the root surface. Spores and hyphae are indicated by orange and white arrows, respectively. Bar equals to 20µm.

Two weeks after *Actinomycetota* inoculation, plants roots were analyzed with SEM to evaluate their colonization intensity. The results are presented in **Figure 1** and show that treated roots surface were covered by *Actinomycetota* strains. This indicated that these strains successfully colonized without damage to the root surface, while those from un-inoculated plants did not. In addition, the mycelial growth penetrating the outer layer of the root as well as sporulation were observed for all the tested strains compared to the un-inoculated controls. In addition, the extent of colonization was more pronounced with *N. alba* strain BC11.

**Figure 2:**
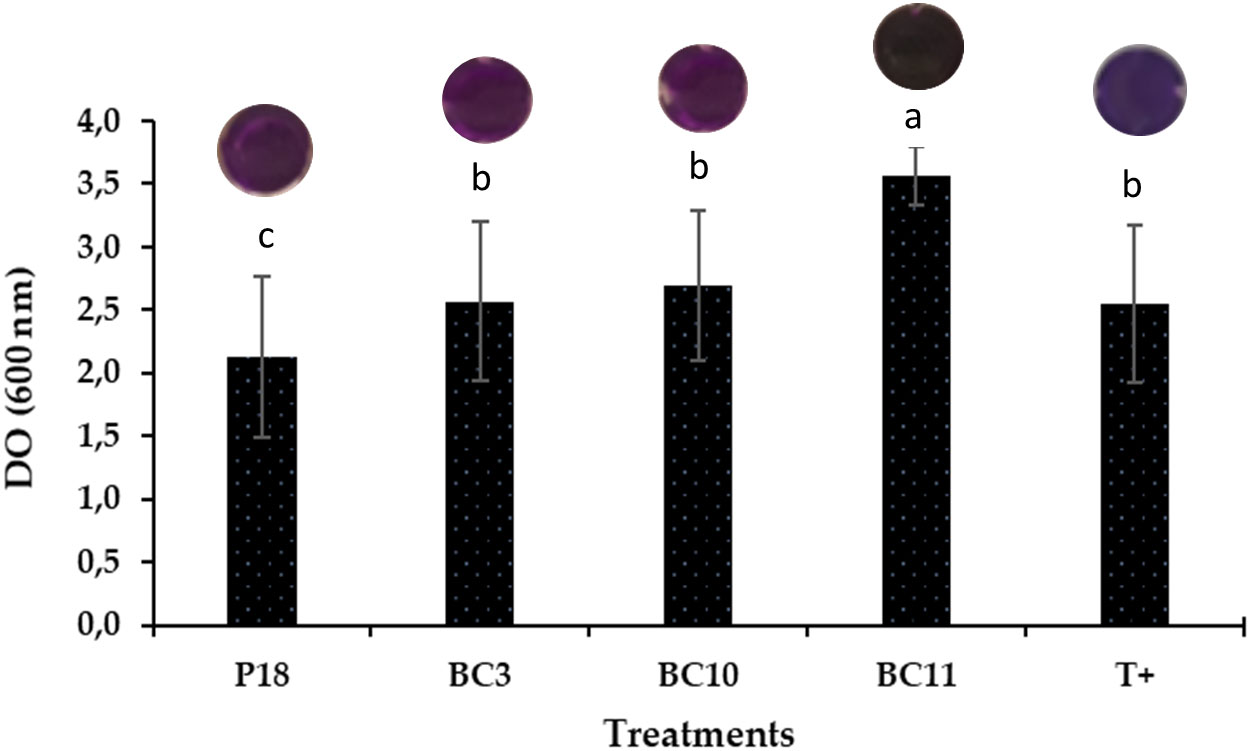
Biofilm formation by the selected *Actinomycetota* strains. The values represent means of replicates (n=3). *Pseudomonas aeruginosa* was used as a positive control C^+^.

On the other hand, the crystal violet binding assay demonstrated a strong biofilm formation in all *Actinomycetota* strains compared to the non-inoculated control expected for *S. anulatus* strain P16. The highest amount was recorded by *N. alba* strain BC11 followed by *S. griseorubens* strain BC3 and *S. griseorubens* strain BC10 (**Figure 2**).

### 3.2 Effect of soil pH-*Actinomycetota* and RPs inoculations on biomass production in wheat

Co-inoculations with the *Actinomycetota* strains and RPs improved the yield and physiological parameters compared to the uninoculated controls and displayed higher values than those inoculated with the RPs alone. The strains were more performant with RP (RP3) and RP (RP4) regardless the type of soil used. In alkaline soil, the highest shoot dry weight (SDW) (+42%), root dry weight (RDW) (+69.5%), and spike biomass (+97%) were recorded by the following treatments: BC3.RP4, P18.RP3, and BC3.RP4 respectively in comparison with their control RPs (**Table 3**). However, in acidic soil, the highest agronomic performances of growth and yield (+124.12%) were recorded for treatments fertilized with RP3 rock. In addition, results have shown that *Actinomycetota*-RP combination were agronomically more efficient in alkaline and acidic soils as compared to positive control, BG4 (**Table 3**).

**Table 3:** Agronomic effectiveness of *Actinomycetota*-RP combination in alkaline/acidic soil compared with BG4 In alkaline soil.

Soil type	Treatments	Shoot dry weight				Root dry weight				Spike biomass			
		RP1	RP2	RP3	RP4	RP1	RP2	RP3	RP4	RP1	RP2	RP3	RP4
**Alkaline soil**	**P16**	–	–	+25%a	+25.2%b	–	+2.188%c	+30.7%e	–	–	–	+35.5%a	+54%c
	**P18**	–	–	+13.8%b	+17%c	+18.5%a	+15.15%b	+69.5%b	–	–	–	+28.2%b	+82%b
	**BC3**	+1.029%b	–	–	+42.8%a	+5.51%b	–	+47.6%d	+7.4%b	+6.43%a	–	–	+97%a
	**BC10**	+5.51%a	–	+4.5%d	+3.8%d	–	+38.58%a	+59%cd	–	+4.02%b	+2.43%b	+26%b	+35.6%d
	**BC11**	–	+11.88%ab	+7%c	+31.36%b	+1.60%c	–	+90%a	+27.43a	+6.70%a	+37.69%a	+23.3%c	+59%c
												
**Acidic soil**	**P16**	–	+9.22%c	+32.50%a	–	+12.31%c	+7.70%a	+13.04%b	–	–	+11.15%c	+29.82%bc	–
	**P18**	+39.29%a	–	+10.89%c	+27.93%a	+28.64%b	–	–	+12.17%d	+76.87%b	–	+6.14%d	+51.85%c
	**BC3**	+39.60%a	+16.86%b	+10%c	+24.72%a	+39.95%a	–	–	+21.95%c	+115%a	+40.28%b	+20.17%c	+59.25%b
	**BC10**	+0.83%c	+8.57%c	+29.14%b	+15.33%b	–	–	–	+30.86%b	+51.87%c	+14.38%c	+124.12%a	+54.62%b
	**BC11**	+28.17%b	+27.4%a	+28.07%b	+8.52%c	+33.91%ab	+5.67%b	+39.95%a	+38.26%a	+110.62%a	+53.6%a	+72.37%b	+80.55%a

Different letters indicate significant differences (p <0.05).

### 3.3 Effect of *Actinomycetota*-RPs combinations on P and K content in plant tissues

**Figure 3:**
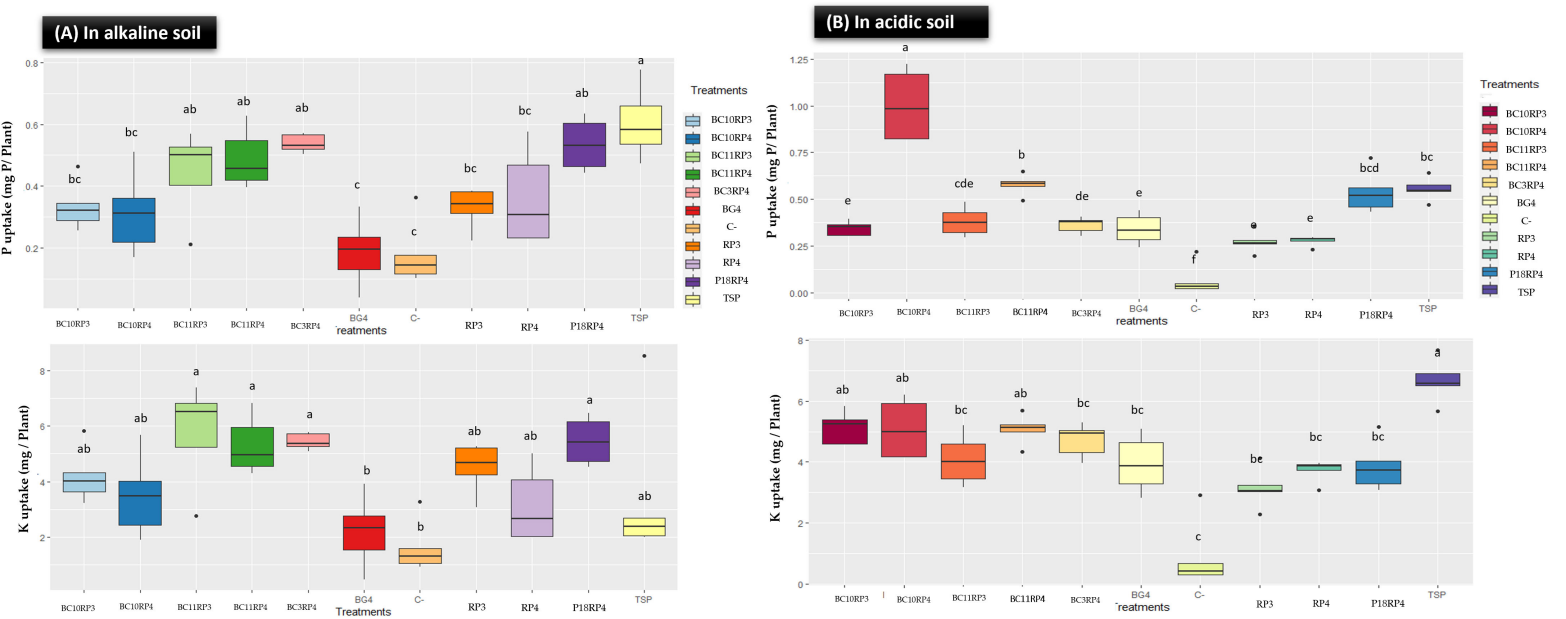
Effect of *Actinomycetota* and RPs combinations on P and K uptakes in wheat plants tissue. **(A)** In alkaline conditions; **(B)** In acidic conditions. Different letters indicate significant differences (p <0.05).

The performance on P and K content in plant tissues of the following combinations BC10.RP3, BC11.RP3, P18.RP4, BC3.RP4, BC10.RP4, and BC11.RP4 are presented in **Figure 3**. A significant improvement in P and K content in wheat plants tissues was noted with the *Actinomycetota*-RP combinations compared to uninoculated treatments (C-) and controls rocks (RP3 and RP4). In fact, P uptake in the shoot increased by 80.10%, 137.63%, 34.9%, 189.78%, 68.81% and 162.90% respectively for BC10.RP3, BC11.RP3, P18.RP4, BC3.RP4, BC10.RP4, and BC11.RP4 treatments as compared to the BG4 (**Figure 3A**). Furthermore, the K content increased by 19.39% to 62.91% for the same treatments as compared to the positive control TSP. In alkaline soil, results showed that negative controls (C^-^) as well as BG4 treatments did not significantly increase the P and K content in wheat plants (**Figure 3A**). In addition, potassium and phosphorus deficiency symptoms (necrosis of the leaf tips or margins and orangish discoloration) were observed on the tips of the leaves for these treatments (data not shown).

In acidic soil, the direct application of *Actinomycetota* and RP significantly increased the P and K content in wheat plants compared to control treatments, reactive rock BG4 (**Figure 3B**). Interestingly, the highest total P content in plant tissues was observed in the treatments *N. alba* strain/BC11.RP4 and *S. griseorubens* strain/BC10.RP4 since they were performant as compared to the BG4 but also increased the P content by 3.96% and 80.75% respectively in comparison with TSP. In general, the amount of P and K content of wheat plants tissues were more pronounced in alkaline soil than acidic soil.

### 3.4 Chlorophyll content

The results summarized in **Table 4** show that the selected strains increased the chlorophyll content in the leaves of wheat plants up to 31.32% and 42.29% in alkaline and acidic conditions respectively, as compared to the use of BG4. The maximum chlorophyll contents were recorded in the plants co-inoculated with *N. alba* strain BC11.RP4 followed by *S. griseorubens* strain BC3.RP4 regardless of soil type used. However, severe or prolonged P deficiency was recorded in the control treatments (RPs and negative controls) which showed a purple/brown leaves.

**Table 4:** Effect of *Actinomycetota*-RPs inoculations on chlorophyll content of wheat plants.

Treatments	Chlorophyll content index	
	Alkaline conditions	Acidic conditions
**C-**	18.53 ± 0.104c	13.26 ± 0.155f
**TSP**	23.199 ± 0.48a	29.71 ± 0.51a
**BG4**	16.83 ± 0.233d	20.31 ± 0.212cd
**RP3**	17.57 ± 0.085cd	15.46 ± 0.314e
**P16RP3**	20.58 ± 0.075b	16.56 ± 0.32de
**P18RP3**	19.82 ± 0.19abc	17.183 ± 1.13d
**BC3RP3**	22.003 ± 0.671ab	22.98 ± 1.44c
**BC10RP3**	21.34 ± 0.09ab	26.27 ± 2.86ab
**BC11RP3**	21.93 ± 0.078ab	22.24 ± 2.94c
**RP4**	20.18 ± 1.57b	18.34 ± 1.75d
**P16RP4**	19.214 ± 0.023abc	18.376 ± 0.16d
**P18RP4**	20.58 ± 1.207b	25.76 ± 1.105abc
**BC3RP4**	22.10 ± 0.635ab	27.326 ± 0.56ab
**BC10RP4**	20.899 ± 1.78ab	26.103 ± 0.196abc
**BC11RP4**	22.101 ± 0.412ab	28.90 ± 0.512ab

Different letters indicate significant differences (p <t0.05).

### 3.5 Determination of residual P and K nutrients in soil

The effect of *Actinomycetota*-RPs on the residual P and K in soil are presented in **Table 5**.

**Table 5:** Effect of *Actinomycetota*-RPs on available P and K in soil.

Soil conditions	Treatments	P (mg/kg)	K (mg/kg)
**Acidic conditions**	**P18.RP4**	0.103 ± 0.011 b	0.742 ± 0.08 cd
	**BC3.RP4**	0.064 ± 0.005 cd	0.836 ± 0.063 bcd
	**BC10.RP4**	0.166 ± 0.018 a	0.842 ± 0.091 bcd
	**BC11.RP4**	0.103 ± 0.008 bc	0.996 ± 0.07 bcd
	**BC10.RP3**	0.08 ± 0.008 bcd	1.188 ± 0.114 a
	**BC11.RP3**	0.061 ± 0.007 d	0.655 ± 0.073 de
	**BG4**	0.083 ± 0.016 bcd	0.963 ± 0.188 abc
	**TSP**	0.089 ± 0.003 bc	1.067 ± 0.035 ab
	**C-**	0.027 ± 0.024 e	0.359 ± 0.323 e
**Alkaline conditions**	**P18.RP4**	0.097 ± 0.019 a	0.987 ± 0.192 a
	**BC3.RP4**	0.089 ± 0.01 ab	0.902 ± 0.096 a
	**BC10.RP4**	0.055 ± 0.016 c	0.607 ± 0.179 ab
	**BC11.RP4**	0.078 ± 0.013 abc	0.741 ± 0.145 ab
	**BC10.RP3**	0.052 ± 0.011 bc	0.649 ± 0.132 ab
	**BC11.RP3**	0.056 ± 0.015 bc	0.732 ± 0.193 ab
	**BG4**	0.05 ± 0.03 c	0.61 ± 0.354 ab
	**TSP**	0.08 ± 0.013 abc	0.44 ± 0.248 b
	**C-**	0.05 ± 0.024 c	0.43 ± 0.220 b

‡Different letters indicate significant differences (p<0.05).

As compared to the BG4, the available P and K in all treatments increased to different levels depending on the type of the soil used. In acidic soil, the following treatments BC10.RP4, P18.RP4 and BC11.RP4 increased the available P from 24.09% to 100% as compared to BG4 and from 15.73% to 86.51% as compared to TSP. However, a maximum of available K was recorded with the treatment fertilized with BC11.RP4 with an increase of 3.63% compared to BG4. On the other hand, when the *Actinomycetota* strains were inoculated in alkaline soil, the combinations P18.RP4, BC3.RP4 and BC11.RP4 were the most performant since they increase up to 86.54% the available P and up to 61.01% the available K in soil compared to BG4.

### 3.6 Correlation and multivariate analysis

**Figure 4:**
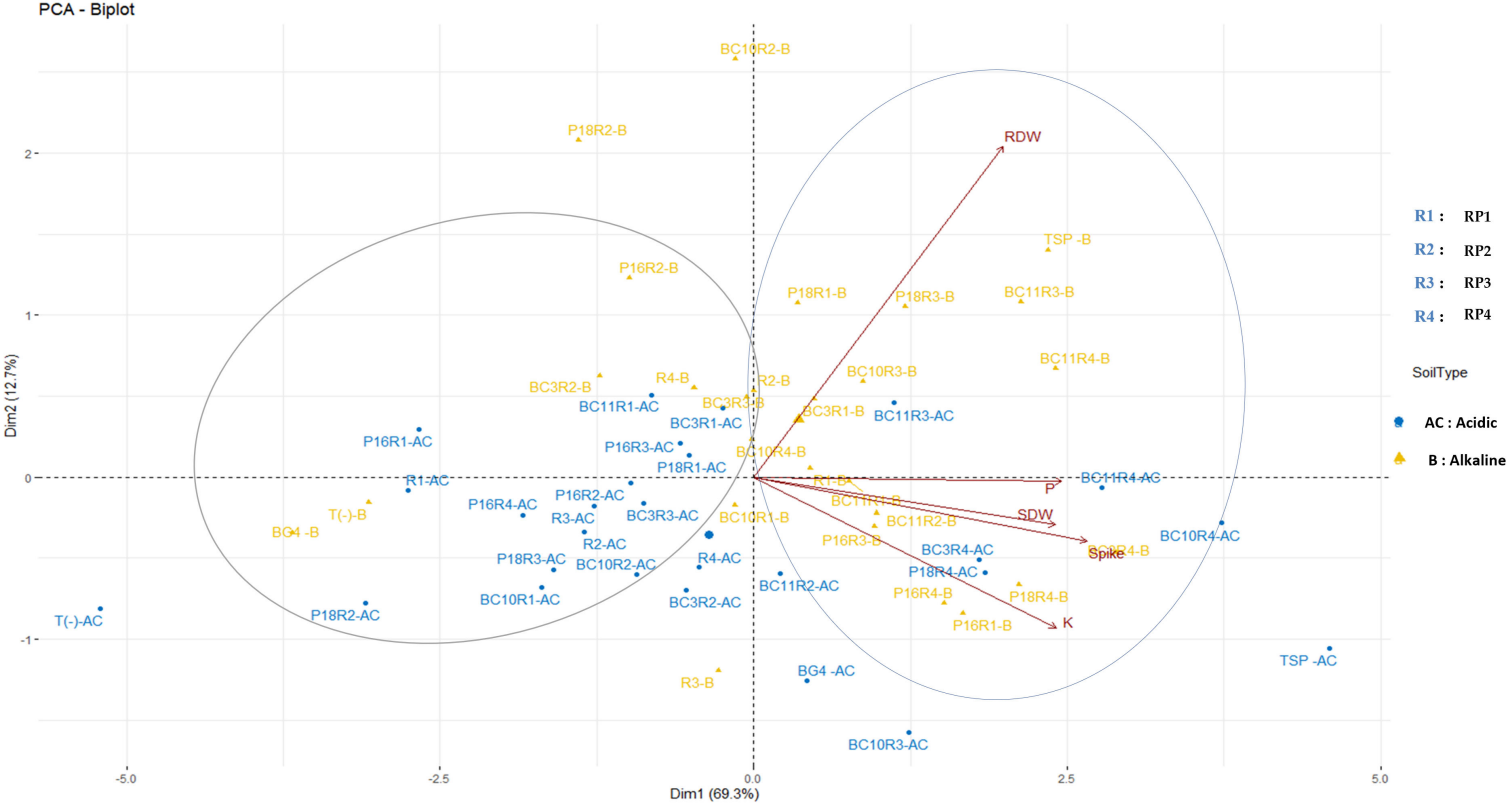
Principal components analysis of the wheat growth parameters and nutrient content across different *Actinomycetota*-RPs inoculations in different soil pH. The points represent mean values of 5 replications of each treatment. Arrows indicate directions and strength of parameters in the dataset. SDW, Shoot dry weight; RDW, Root Dry weight.

According to the PCA analysis (**Figure 4**), the two principal components (Dim1 and Dim2) account for 82% of the total variation. The variation in the data is maximal with first axis accounting for 69,3% followed by the second axis (12,7% of the variance). Following this two first axis, the data are grouped into two major clusters. The first cluster consists of treatments that significantly increase the nutritional and agronomic parameters. However, the second group summarized the less efficient treatments that have a negative correlation with the tested parameters. In acidic soil, the most efficient treatments follow each other in this order: P18.RP4, BC3.RP4, BC10.RP4, BC11.RP3, and BC11.RP4 whereas in alkaline soil the order is as follow: P16.RP3, P18.RP3, BC10.RP3, BC11.RP3, BC10.RP4, BC11.RP4, BC3.RP4, P18.RP4, P16.RP4 and P16.RP1. These findings showed a clear separation between the fertilization under alkaline and acidic conditions.

The P-values of the MANOVA analysis between the different interactions revealed significant interactions (*p<0.001*) between RPs and *Actinomycetota* as well as the interactions between Soil, RPs, and *Actinomycetota* for all the measured agronomic parameters (**Supplementary Data Table 1**). However, the relationship between soil pH, Spike, P content, and K content in wheat plant tissues was found to be non-significant which confirms the poor availability of nutrients in the soils used in this study.

Furthermore, the agronomic parameters (SDW, RDW, Spike) and nutrient content (P and K) of plant tissues were significantly correlated except for RDW which was weakly correlated with P content (R^2^ = 0.270, p<0.001) and K content (R^2^ = 0.161) (**Supplementary Data Table 2**).

### 3.7 Effect size analysis

The treatments effects in this experiment are generally so pronounced when the plants were grown in alkaline soil than acidic soil. In alkaline conditions, we only needed between 2 to 5 experimental units in each treatment to achieve 90% power except the parameters P and K content (**Table 6**). The larger sample sizes used provide additional power for making multiple comparisons between treatments, ranging from 0.90 to 1. These coefficients are judged to be high size by Cohen (1988) guidelines. For acidic soil, the size of the effect so pronounced but more replications are required in particular spike, SWD, and RDW.

**Table 6:** Effect size analysis.

Soil type	Treatments	Factors	Size for 0.90 power	Actual size	Power for actual size
**Acidic soil**	**BC10RP3**	SDW	10-11	5	0.55
		RDW	5	5	1
		Spike	12-13	5	0.45
		P content	4-5	5	0.9999988
		K content	5	5	1
	**BC11RP3**	SDW	9-10	5	0.62
		RDW	3-4	5	0.9999731
		Spike	3-4	5	0.9999649
		P content	3-4	5	0.9999813
		K content	2	5	0.9990435
	**P18RP4**	SDW	9-10	5	0.62
		RDW	5-6	5	1
		Spike	12-13	5	0.45
		P content	5	5	1
		K content	2-3	5	0.9971115
	**BC3RP4**	SDW	14-15	5	0.40
		RDW	7-8	5	0.66
		Spike	10-11	5	0.55
		P content	4-5	5	1
		K content	4-5	5	1
	**BC10RP4**	SDW	8	5	0.66
		RDW	6-7	5	1
		Spike	10-11	5	0.64
		P content	7-8	5	1
		K content	4-5	5	0.9999933
	**BC11RP4**	SDW	6-7	5	1
		RDW	10	5	0.56
		Spike	10	5	0.55
		P content	8	5	0.78
		K content	4-5	5	1
**Alkaline soil**	**BC10RP3**	SDW	3	5	0.9995942
		RDW	4-5	5	1
		Spike	4-5	5	1
		P content	7-8	5	0.7391821
		K content	3	5	0.9866806
	**BC11RP3**	SDW	2-3	5	0.9899316
		RDW	2	5	1
		Spike	2-3	5	0.9904394
		P content	4-5	5	0.9091433
		K content	2-3	5	0.993249
	**P18RP4**	SDW	5	5	0.9427235
		RDW	5	5	1
		Spike	5	5	1
		P content	4-5	5	0.999953
		K content	4-5	5	0.999999
	**BC3RP4**	SDW	2-3	5	0.9209139
		RDW	5	5	1
		Spike	5	5	0.9999999
		P content	5	5	0.9999999
		K content	5	5	1
	**BC10RP4**	SDW	3-4	5	0.9801401
		RDW	2-3	5	0.9873727
		Spike	4-5	5	0.9999926
		P content	17	5	0.4199026
		K content	9	5	0.6613789
	**BC11RP4**	SDW	2	5	0.9989766
		RDW	2	5	1
		Spike	4-5	5	0.9999995
		P content	2-3	5	0.9977018
		K content	2	5	0.9999378

## 4 Discussion

This study has demonstrated that the PSA effectively colonized the wheat root surface and formed a strong biofilm along epidermal tissues. This close interactions confers the *Actinomycetota* strains an advantage to influence positively wheat growth, and yield (Merzaeva and Shirokikh, 2006; Goudjal et al., 2016; van der Meij et al., 2017). Our results are consistent with the findings of Mun et al. (2020) that reported successful colonization of cucumber root by *Streptomyces* LH4 and suggested that this phenomenon may produce a staple effect by LH4 on the growth and defense system of the plant. Moreover, it has been reported that biofilm formation is considered a protective mechanism that is an additional advantage for plants that safeguard them from external stresses and microbial competition (Wu et al., 2019). The greenhouse experiments demonstrated that the agronomic performances of the *Actinomycetota* combined with RPs were greatly influenced by RP grades, soil characteristics and soil pH (**Table 3**). The high significant effect size indicate that our experiment is more likely to lead to conclusive results as previously highlighted by Soumare et al. (2015). In our study, the grade RP4 and RP3 containing the highest P_2_O_5_ content (32.81% and 31.12% respectively), has resulted in the best agronomic performance of wheat plant. Similar studies have been reported by Xiao et al. (2008) and Gomes et al. (2014) who have shown that the solubilization capacity of microorganisms was also correlated positively with the grade of RP. In this regard, our previous results in *in-vitro* screening on NBRIP medium and in greenhouse with maize plants showed the same trends (Soumare et al., 2020a; Boubekri et al., 2021). Even though *Actinomycetota* strains could solubilize the RPs in both acid and alkaline soil type as it has been demonstrated in this study, the agronomic performances on wheat plants were more pronounced in alkaline soil than acidic soil. The results obtained are in accordance with those of Alam et al. (2022) who observed a marked increase in all agronomic parameters of wheat when mineral P was applied along with PSB in alkaline soil. Our findings are also consistent with those of Amaresan et al. (2020) who have shown that *Actinomycetota* grew much better in the pH 6.0 to 9.0 range than in a more acidic or alkaline soil. In this regard, the highest shoot dry weight (+42% compared to RP4) and root dry weights (+69.5% compared to RP3) were always recorded when the wheat was planted in alkaline soil (**Table 3**). Moreover, the combined application of *Actinomycetota* resulted in higher spike yield of 19.7% in alkaline soil (*N. alba* BC11.RP4) and 4.97% in acidic soil (*S. griseorubens* BC10.RP3) compared to the TSP treatment (**Table 3**). In fact, the combined application of RP with soil microorganisms is like a slow release biofertilizer which reduce the P leaching in soil, which bring continuously the available nutrients to plants (Wang et al., 2020). Indeed, if P is available in large quantities as for TSP, it is subjected to leaching, complexation with either calcium or aluminium (Bouray et al., 2021). Interestingly, our findings highlight that the strains are competitive with the native flora and there was no antagonism between the inoculated *Actinomycetota* and the native microorganisms since they effectively improved the wheat plant growth under non-sterile substrate. The important influence of soil pH on the performances of the *Actinomycetota*-RPs combinations has been confirmed as our previous studies. It has been reported that soil pH influences the microorganism activity and nutrients solubility, thereby, affecting the growth and yields of plants (Gondal et al., 2021). P availability and mobility are low in most soils, especially in acidic soils where P availability is mainly limited by adsorption reactions due to low pH and high concentrations of aluminum and iron oxides and hydroxides (Penn and Camberato, 2019). For instance, in acidic soils the plant growth is favored because most micronutrients are more available to plants than in neutral-alkaline soils.

In addition to soil pH, soil texture is also thought to be a key factor affecting nutrient’s availability especially for P and K (McLauchlan, 2006; Fageria and Moreira, 2011; Soumare et al., 2022). In fact, results revealed that the agronomic performances of wheat plants of clay-loamy soil are significantly different from that of sandy soil. The highest performance was found in clay-loamy soil than sandy soil is probably due to the high of water retention and nutrient-holding capacities that are necessary for plant growth. In sandy soil, the fine particles allows rapid leaching of nutrients from soil (Carrenho et al., 2007; Afzal et al., 2011; Ouzounidou et al., 2015). Our findings corroborate those of Egamberdiyeva (2007) and Islam et al. (2018) who demonstrated a better stimulatory effect of PSB in loamy soil than sandy soil. The increment of chlorophyll content is considered to be a parameter which corresponds to an increase in photosynthesis, and, consequently, to an increase in production potential and plant vigor (Bashan et al., 2006; Pereira et al., 2015). These results demonstrate the contribution of tested strains (especially *N. alba* strain BC11with RP4 followed by *S. griseorubens* strain BC3 with RP3) to plants P nutrition and photosynthesis. In contrast, the treatments with prolonged P- deficiency (control RPs and negative controls) showed a purples/brown leaves which may result in the accumulation of anthocyanins, consequently increasing the pigmentation of the newest leaves and chlorophyll concentrations (Veazie et al., 2020). This may be due to the greater solubilization/mobilization of P in wheat plants which later, in turn, promotes N content in plants (Adhikari et al., 2021). Plant nutrient status also changes with the different Actinomycetota-RPs combinations in both acidic and alkaline soils. In general, the inoculation of PSA significantly compensated the nutrient deficiency especially P by stimulating root development which led to a better adsorption of water and nutrients. Indeed, it has been found that the addition of *Actinomycetota* bio-inoculants along with RP fertilizations were able to reverse the low level of P and K assimilation and accumulation observed in the stems of negative controls and RPs controls, reaching P assimilation levels similar to those observed in the positive controls fertilized with TSP. The main reason could be due to increased P and K availability in soil which is latter utilized by the wheat plant itself for growth upon PSA inoculation. These results were supported by Swarnalakshmi et al. (2013), where combined application of PSB and RP significantly promotes wheat plant P content in comparison with the mineral fertilizers or with the single PSB inoculation. Similar results have been reported by Dasila et al. (2023) who demonstrated that PSB inoculation significantly improve the nutrition status of the wheat plants.

In the present study, the highest increase in total P and K content in plant tissues was observed in *N. alba* strain BC11.RP4 with an improvement up to 162.9% and 142.53% respectively in alkaline conditions compared to BG4. In addition, the inoculation with the BC10.RP4 and BC11.RP4 increased the P content in soil by more than 15.73% compared to the TSP. However, under acidic conditions, BC10.RP4 followed by *N. alba* strain BC11.RP4 were the most performant inoculums in terms of increasing the nutrient uptake since the latter increased by up to 195.58% in the case of P and 29.56% in the case of K compared to BG4. This observation provides an explanation that inoculation of PSA promotes oil nutrient status *via* solubilizing/mobilizing soil nutrients. These findings were in tune with the studies of Hamdali et al. (2012); Biglari et al. (2016); El-Badan et al. (2019); Vargas Hoyos et al. (2021) who demonstrated that application of RP with *Actinomycetota* strains enriched the rhizosphere with soil available P compared to other treatments. Therefore, the increase of the P and K availability under *Actinomycetota*-RP fertilization suggest also that the inoculated bacterial strains positively compete with existing natural bacteria. In addition, the Manova analysis revealed significant interactions (*p<0.001*) between soil*RP*Actinomycetota and the agronomic parameters of wheat plants which explained their synergic effects (**Supplementary Data Table 1**). Thus, these results are in line with those reported by Mittal et al. (2008) and Sharma et al. (2013) that have shown that the in addition to the yield and wheat nutrient uptake improvement obtained by the application of rock phosphate with PSB, the subsequent crop will reap the benefits impaired by the PSB to the soils. Finally, these findings suggest that the *N. alba* strain BC11 is a valuable resource for sustainable agriculture and could help alleviate agricultural losses due to P limitation in acid and alkaline soils while maintaining and improving yields.

## 5 Conclusion

This first report of combining Actinomycetota-RP application to promote wheat growth under natural alkaline and acidic soils clearly indicated that the tested PSA are able to solubilize a broad spectrum of RPs, but their efficiencies depend on RP grades, soil pH, and soil type. Regardless of the soil type used, PSA along with RP3/RP4 showed similar or high performance as compared to the positive controls BG4 and TSP. This increase is due to their ability to solubilize a broad spectrum of RP, to effectively colonize the wheat root systems, to form a strong biofilm as well as their capacity to produce plant growth promoting factors. Amongst the PSA, *N. alba* strain BC11 along with RP4, was effective in optimizing wheat yield attributes especially in alkaline soil. This reveals the potential of this strain for biofertilizer applications and its potential for sustainable agriculture and environment. Combined application of *Actinomycetota* and RP is therefore an emerging option for meeting agricultural challenges and providing an excellent opportunity to develop environment-friendly phosphorus biofertilizer adapted for P-deficient alkaline and acidic soils. The positive outcome of this investigation shall be verified in field conditions under diverse agro-climatic regions on a variety of crops. Prior to recommend the suggested biofertilizer, supplementary research is needed such as: optimizing the biofertilizer formulation, evaluate its shelf-life, and conduct a market study for future commercialization.

## Data availability statement

The original contributions presented in the study are included in the article/**Supplementary Material**. Further inquiries can be directed to the corresponding authors.

## Author contributions

KB: Conceptualization, investigation, methodology, formal analysis, software, writing original draft. AS: conceptualization, investigation, methodology, formal analysis, writing original draft, review and editing. KL: Supervision, review and editing. YO: Supervision, review and editing. MH: Supervision, review and editing. LK: Project administration, validation, supervision, review and editing. All authors contributed to the article and approved the submitted version.

## References

[B1] Adhikari P. Jain R. Sharma A. Pandey A. (2021). Plant growth promotion at low temperature by phosphate solubilizing pseudomonas spp. isolated from high-altitude Himalayan soil. Microb. Ecol. 82 (3), 677–687. doi: 10.1007/s00248-021-01702-1 33512536

[B2] Afzal M. Yousaf S. Reichenauer T. G. Kuffner M. Sessitsch A. (2011). Soil type affects plant colonization, activity and catabolic gene expression of inoculated bacterial strains during phytoremediation of diesel. J. Hazard. Mater. 186 2–3, 1568–1575. doi: 10.1016/j.jhazmat.2010.12.040 21216097

[B3] Alam F. Khan A. Fahad S. Nawaz S. Ahmed N. Ali M. A.. (2022). Phosphate solubilizing bacteria optimize wheat yield in mineral phosphorus applied alkaline soil. J. Saudi Soc Agric. Sci. 21 (5), 339–348. doi: 10.1016/j.jssas.2021.10.007

[B4] Amaresan N. Kumar M. S. Annapurna K. Kumar K. Sankaranaryanan N. (2020). Beneficial microbes in agro-ecology: bacteria and fungi (Academic Press).

[B5] Anand U. Pal T. Yadav N. Singh V. K. Tripathi V. Choudhary K. K. (2023). Current scenario and future prospects of endophytic microbes: promising candidates for abiotic and biotic stress management for agricultural and environemental sustainability. Microb. Ecol., 1–32. doi: 10.1007/s00248-023-02190-1 PMC10497456 36917283

[B6] Arcand M. M. Schneider K. D. (2006). Plant- and microbial-based mechanisms to improve the agronomic effectiveness of phosphate rock: a review. An. Acad. Bras. Cienc. 78 (4), 791–807. doi: 10.1590/S0001-37652006000400013 17143413

[B7] Bashan Y. Bustillos J. J. Leyva L. A. Hernandez J. P. Bacilio M. (2006). Increase in auxiliary photoprotective photosynthetic pigments in wheat seedlings induced by azospirillum brasilense. Biol. Fertil. Soils. 42 (4), 279–285. doi: 10.1007/s00374-005-0025-x

[B8] Bhatti A. A. Haq S. Bhat R. A. (2017). Actinomycetes benefaction role in soil and plant health. Microb. Pathog. 111, 458–467. doi: 10.1016/j.micpath.2017.09.036 28923606

[B9] Biglari N. Hassan H. M. Amini. J. (2016). The ability of streptomyces spp. isolated from Iranian soil to solubilize rock phosphate. ABCmed. 4 (3), 15–25. doi: 10.7575/aiac.abcmed.16.04.03.03

[B10] Boubekri K. Soumare A. Mardad I. Lyamlouli K. Hafidi M. Ouhdouch Y.. (2021). The screening of potassium-and phosphate-solubilizing *Actinomycetota* and the assessment of their ability to promote wheat growth parameters. Microorganisms. 9 (3), 1–16. doi: 10.3390/microorganisms9030470 PMC7996285 33668691

[B11] Boubekri K. Soumare A. Mardad I. Lyamlouli K. Ouhdouch Y. Hafidi M.. (2022). Multifunctional role of *Actinomycetota* in agricultural production sustainability : a review. Microbiol. Res., 127059. doi: 10.1016/j.micres.2022.127059 35584559

[B12] Bouray M. Moir J. L. Lehto N. J. Condron L. M. Touhami D. Hummel C. (2021). Soil pH effects on phosphorus mobilization in the rhizosphere of lupinus angustifolius. Plant Soil. 469 (1–2), 387–407. doi: 10.1007/s11104-021-05177-4

[B13] Bringel J. M. M. (1997). Colonizaçao de raızes de plantas cultivadas por pseudomonas solanacearum biovares 1, 2 e 3 em condiçoes de casa de vegetaçao e ÔÔin vitroÕÕ (Doctoral dissertation, master dissertation, depto. fitopatologia, universidade de brasılia). root colonization potential of Actinomycetota strains section

[B14] Carrenho R. Trufem S. F. B. Bononi V. L. R. Silva E. S. (2007). The effect of different soil properties on arbuscular mycorrhizal colonization of peanuts, sorghum and maize. Acta Bot. Bras. 21 (3), 723–730. doi: 10.1590/S0102-33062007000300018

[B15] Chaudhry V. Runge P. Sengupta P. Doehlemann G. Parker J. E. Kemen E. (2021). Shaping the leaf microbiota: plant microbe-microbe interactions. J. Exp. Bot. 72 (1), 36–56. doi: 10.1093/jxb/eraa417 32910810 PMC8210630

[B16] Christensen G. D. Simpson W. A. Younger J. J. Baddour L. M. Barrett F. F. Melton D. M.. (1985). Adherence of coagulase-negative staphylococci to plastic tissue culture Plates : a quantitative model for the adherence of staphylococci to medical devices. J. Clin. Microbiol. 22 (6), 996–1006. doi: 10.1128/jcm.22.6.996-1006.1985 3905855 PMC271866

[B17] Cohen J. (1988). Statistical power analysis for the behavioral sciences. 2nd edn (Hillsdale, NJ: Lawrence Erlbaum Associates Inc).

[B18] Dasila H. Sah V. K. Jaggi V. Kumar A. Tewari L. Taj G.. (2023). Cold tolerant phosphate solubilizing pseudomonas strains promote wheat growth and yield by improving soil phosphorus (P) nutrition status. Front. Microbiol. 14. doi: 10.3389/fmicb.2023.1135693 PMC10072159 37025630

[B19] de Oliveira C. A. Gomes U. Lana U. G. D. P. (2014). Rock phosphate solubilizing microorganisms isolated from maize rhizosphere soil. Rev. Bras. Milho sorgo. 13, 69–81. doi: 10.18512/1980-6477/rbms.v13n1p69-81

[B20] Egamberdiyeva D. (2007). The effect of plant growth promoting bacteria on growth and nutrient uptake of maize in two different soils. Appl. Soil Ecol. 36, 184–189. doi: 10.1016/j.apsoil.2007.02.005

[B21] El-Badan D. E. Hala H. B. Hanan M. Hanan G. Soraya A. F. E. (2019). Evaluation for rock phosphate solubilization using streptomyces sp.RHS33. Adv. Appl. Microbiol. 1 (1), 1–11.

[B22] El-Tarabily K. A. ElBaghdady K. Z. AlKhajeh A. S. Ayyash M. M. Aljneibi R. S. El-Keblawy A.. (2020). Polyamine-producing *Actinomycetota* enhance biomass production and seed yield in salicornia bigelovii. Biol. Fertil. Soils. 56 (4), 499–519. doi: 10.1007/s00374-020-01450-3

[B23] Fageria N. K. Moreira A. (2011). The role of mineral nutrition on root growth of crop plants. Adv. Agron. 110, 251–331. doi: 10.1016/B978-0-12-385531-2.00004-9

[B24] Fageria N. K. Nascente A. S. (2014). Management of soil acidity of south American soils for sustainable crop production. Adv. Agron. 128, 221–275. doi: 10.1016/B978-0-12-802139-2.00006-8

[B25] Fahsi N. Mahdi I. Mesfioui A. Biskri L. Allaoui A. (2021). Plant growth-promoting rhizobacteria isolated from the jujube (Ziziphus lotus) plant enhance wheat growth, zn uptake, and heavy metal tolerance. Agriculture. 11 (4), 316. doi: 10.3390/agriculture11040316

[B26] Gomes E. A. Silva U. D. C Marriel I. E. De Oliveira C.A. Lana U. G. D. P. (2014). Rock phosphate solubilizing microorganisms isolated from maize rhizosphere soil. Rev. Bras. Milho sorgo. 13, 69–81. doi: 10.18512/1980-6477/rbms.v13n1p69-81

[B27] Gondal A. H. Hussain I. Ijaz A. B. Zafar A. Ch B. I. Zafar H.. (2021). Influence of soil pH and microbes on mineral solubility and plant nutrition: a review. Int. J. Agric. Biol. 5 (1), 2–12.

[B28] Gopalakrishnan S. Srinivas V. Alekhya G. Prakash B. Kudapa H. Varshney R. K. (2015). Evaluation of broad-spectrum streptomyces sp. for plant growth promotion traits in chickpea (Cicer arietinum l.). Philipp. Agric. Sci. 98 (3), 270–278.

[B29] Goudjal Y. Zamoum M. Meklat A. Sabaou N. Mathieu F. Zitouni A. (2016). Plant-growth-promoting potential of endosymbiotic *Actinomycetota* isolated from sand truffles (Terfezia leonis tul.) of the Algerian Sahara. Ann. Microbiol. 66 (1), 91–100. doi: 10.1007/s13213-015-1085-2

[B30] Hamdali H. Bouizgarne B. Hafidi M. Lebrihi A. Virolle M. J. Ouhdouch Y. (2008). Screening for rock phosphate solubilizing actinomycetes from Moroccan phosphate mines. Appl. Soil Ecol. 38 (1), 12–19. doi: 10.1016/j.apsoil.2007.08.007

[B31] Hamdali H. Moursalou K. Tchangbedji G. Ouhdouch Y. Hafidi M. (2012). Isolation and characterization of rock phosphate solubilizing *Actinomycetota* from Togolese phosphate mine. Afr. J. Biotechnol. 11 (2), 312–320. doi: 10.5897/AJB11.774

[B32] Hellal F. El-Sayed S. Zewainy R. Amer A. (2019). Importance of phosphate pock application for sustaining agricultural production in Egypt. Bull. Natl. Res. Centre 43 (1), 1–11. doi: 10.1186/s42269-019-0050-9

[B33] Islam M. S. Sarker N. R. Md.Y. A. Md.A. H. Billah M. Uddin M. S.. (2018). Impact of loamy and sandy soils on productive and nutritive value of BLRI developed Napier-4 fodder at third cutting stage. Int. J. Appl. Eng. Res. 1 (I), 79–86.

[B34] Kalayu G. (2019). Phosphate solubilizing microorganisms: promising approach as biofertilizers. Int. J. Agron. 2019). doi: 10.1155/2019/4917256

[B35] Mahdi I. Fahsi N. Hafidi M. Benjelloun S. Allaoui A. Biskri L. (2021a). Rhizospheric phosphate solubilizing bacillus atrophaeus GQJK17 S8 increases quinoa seedling, withstands heavy metals, and mitigates salt stress. Sustainability. 13 (6).3307 doi: 10.3390/su13063307

[B36] Mahdi I. Hafidi M. Allaoui A. Biskri L. (2021b). Halotolerant endophytic bacterium serratia rubidaea ED1 enhances phosphate solubilization and promotes seed germination. Agriculture. 11 (3), 224. doi: 10.3390/agriculture11030224

[B37] McLauchlan K. K. (2006). Effects of soil texture on soil carbon and nitrogen dynamics after cessation of agriculture. Geoderma. 136 (1–2), 289–299. doi: 10.1016/j.geoderma.2006.03.053

[B38] Merzaeva O. V. Shirokikh I. G. (2006). Colonization of plant rhizosphere by actinomycetes of different genera. Microbiology. 75 (2), 226–230. doi: 10.1134/S0026261706020184 16758877

[B39] Miranda E. F. O. (1997). Colonização de raízes de plantas daninhas por ralstonia solanacearum" in vitro" e em casa-de-vegetação (Brası´lia, DF – Brasil: Master Dissertation, Depto. Fitopatologia, Universidade de Brası´- lia (UnB).

[B40] Mittal V. Singh O. Nayyar H. Kaur J. Tewari R. (2008). Stimulatory effect of phosphate-solubilizing fungal strains (Aspergillus awamori and penicillium citrinum) on the yield of chickpea (Cicer arietinum l. cv. GPF2). Soil Biol. Biochem. 40 (3), 718–727. doi: 10.1016/j.soilbio.2007.10.008

[B41] Mowafy A. M. S.Agha M. A.Haroun S. A.Abbas M. Elbalkini M. (2022). Insights in nodule-inhabiting plant growth promoting bacteria and their ability to stimulate vicia faba growth. Egypt. J. basic Appl. Sci. 9 (1), 51–64. doi: 10.1080/2314808X.2021.2019418

[B42] Mun B. G. Lee W. H. Kang S. M. Lee S. U. Lee S. M. Lee D. Y.. (2020). Streptomyces sp. LH 4 promotes plant growth and resistance against sclerotinia sclerotiorum in cucumber *via* modulation of enzymatic and defense pathways. Plant Soil. 448:87–103. doi: 10.1007/s11104-019-04411-4

[B43] Murphy P. Riley J. (1962). A modified single solution method for the determination of phopshate in natural waters. Anal. Chim. Acta 27, 31–36. doi: 10.1057/9781137461131

[B44] Neina D. (2019). The role of soil pH in plant nutrition and soil remediation. Appl. Environ. Soil Sci. 2019, 1–9. doi: 10.1155/2019/5794869

[B45] Nicol G. Nicol G. W. Leininger S. Schleper C. Prosser J. I. (2008). The influence of soil pH on the diversity , abundance and transcriptional activity of ammonia oxidizing archaea and bacteria. Environ. Microbiol. 10 (11), 2966–2978. doi: 10.1111/j.1462-2920.2008.01701.x 18707610

[B46] Numan M. Bashir S. Khan Y. Mumtaz R. Shinwari Z. K. Khan A. L.. (2018). Plant growth promoting bacteria as an alternative strategy for salt tolerance in plants: a review. Microbiol. Res. 209, 21–32. doi: 10.1016/j.micres.2018.02.003 29580619

[B47] Olsen S. R. (1954). Estimation of available phosphorus in soils by extraction with sodium bicarbonate (U.S.Department of Agriculture).

[B48] Ouzounidou G. Skiada V. Papadopoulou K. K. Stamatis N. Kavvadias V. Eleftheriadis E.. (2015). Effects of soil pH and arbuscular mycorrhiza (AM) inoculation on growth and chemical composition of chia (Salvia hispanica l.) leaves. Rev. Bras. Bot. 38 (3), 487–495. doi: 10.1007/s40415-015-0166-6

[B49] Pathak R. Paudel V. Shrestha A. Lamichhane J. Gauchan D. P. (2017). Isolation of phosphate solubilizing bacteria and their use for plant growth promoting in tomato seedling and plant. Kathmandu Univ. J. Sciencce Eng. Technology. 13 (2), 61–70. doi: 10.3126/kuset.v13i2.21284

[B50] Penn C. J. Camberato J. J. (2019). A critical review on soil chemical processes that control how soil ph affects phosphorus availability to plants. Agriculture. 9 (6), 1–18. doi: 10.3390/agriculture9060120

[B51] Pereira L. Pereira E. Revolti L. T. M. Zingaretti S. M. Môro G. V. (2015). Seed quality, chlorophyll content index and leaf nitrogen levels in maize inoculated with azospirillum brasilense. Cienc Agron. 46 (3), 630–637. doi: 10.5935/1806-6690.20150047

[B52] Ringeval B. Augusto L. Monod H. Van Apeldoorn D. Bouwman L. Yang X.. (2017). Phosphorus in agricultural soils: drivers of its distribution at the global scale. Glob Chang Biol. 23 (8), 3418–3432. doi: 10.1111/ijlh.12426 28067005

[B53] Sharma S. B. Sayyed R. Z. Trivedi M. H. Gobi T. A. (2013). Phosphate solubilizing microbes: sustainable approach for managing phosphorus deficiency in agricultural soils. SpringerPlus. 2 (12), 1–14. doi: 10.1186/2193-1801-2-587 25674415 PMC4320215

[B54] Shenoy V. V. Kalagudi G. M. (2005). Enhancing plant phosphorus use efficiency for sustainable cropping. Biotechnol. Adv. 23 (7–8), 501–513. doi: 10.1016/j.biotechadv.2005.01.004 16140488

[B55] Soumare A. Boubekri K. Lyamlouli K. Hafidi M. Ouhdouch Y. Kouisni L. (2020a). Efficacy of phosphate solubilizing *Actinomycetota* to improve rock phosphate agronomic effectiveness and plant growth promotion. Rhizosphere. 100284. doi: 10.1016/j.rhisph.2020.100284

[B56] Soumare A. Boubekri K. Lyamlouli K. Hafidi M. Ouhdouch Y. Kouisni L. (2020b). From isolation of phosphate solubilizing microbes to their formulation and use as biofertilizers: status and needs. Front. Bioeng. Biotechnol. 7. doi: 10.3389/fbioe.2019.00425 PMC6962098 31998701

[B57] Soumare A. Manga A. Fall S. Hafidi M. Ndoye I. Duponnois R. (2015). Effect of eucalyptus camaldulensis amendement on soil chemical properties, enzymatic activity, acacia aspecies growth and root symbioses. Agroforest. Syst. 89 (1), 97–106. doi: 10.1007/s10457-014-9744z

[B58] Soumare A. Sarr D. Diédhiou A. G. (2022). ). potassium sources, microorganisms, and plant nutrition-challenges and future research directions: a review. Pedosphere. doi: 10.1016/j.pedsph.2022.06.025

[B59] Souza R. Ambrosini A. Passaglia L. M. P. (2015). Plant growth-promoting bacteria as inoculants in agricultural soils. Genet. Mole. Res. 38, 401–419. doi: 10.1590/S1415-475738420150053 PMC4763327 26537605

[B60] Swarnalakshmi K. Prasanna R. Kumar A. Pattnaik S. Chakravarty K. Shivay Y. S.. (2013). Evaluating the influence of novel cyanobacterial biofilmed biofertilizers on soil fertility and plant nutrition in wheat. Eur. J. Soil Biol. 55, 107–116. doi: 10.1016/j.ejsobi.2012.12.008

[B61] van der Meij A. Willemse J. Schneijderberg M. Geurts R. Raaijmakers J. van Wezel G. (2017). Inter- and intracellular colonization of arabidopsis roots by endophytic *Actinomycetota* and the impact of plant hormones on their antimicrobial activity. Antonie Van Leeuwenhoek. 111 (5), 679–690.222844. doi: 10.1101/222844 PMC5913384 29335919

[B62] Vargas Hoyos H. A. Chiaramonte J. B. Barbosa-Casteliani A. G. Fernandez Morais J. Perez-Jaramillo J. E. Nobre Santos S.. (2021). An actinobacterium strain from soil of cerrado promotes phosphorus solubilization and plant growth in soybean plants. Fronti. Bioeng. Biotechnol. 9. doi: 10.3389/fbioe.2021.579906 PMC8100043 33968908

[B63] Veazie P. Cockson P. Henry J. Perkins-Veazie P. Whipker B. (2020). Characterization of nutrient disorders and impacts on chlorophyll and anthocyanin concentration of brassica rapa var. chinensis. Agriculture. 10 (10), 461. doi: 10.3390/agriculture10100461

[B64] Veneklaas E. J. Lambers H. Bragg J. Fineegan P. M. Lovelock C. E. Plaxton W. C.. (2012). Opportunities for improving phosphorus-use efficiency in crop plants. New Phytol. 195 (2), 306–320. 22691045 10.1111/j.1469-8137.2012.04190.x

[B65] Vuuren D. Bouwman A. F. Beusen A. H. W. (2010). Phosphorus demand for the 1970 – 2100 period : a scenario analysis of resource depletion. Glob. Environ. Change 20 (3), 428–439. doi: 10.1016/j.gloenvcha.2010.04.004

[B66] Wahid F. Fahad S. Danish S. Adnan M. Yue Z. Saud S.. (2020). Sustainable management with mycorrhizae and phosphate solubilizing bacteria for enhanced phosphorus uptake in calcareous soils. Agriculture. 10 (8), 334. doi: 10.3390/agriculture10080334

[B67] Wang X. Xiong J. He Z. (2020). Activated dolomite phosphate rock fertilizers to reduce leaching of phosphorus and trace metals as compared to superphosphate. J. Environ. Manage. 255, 109872. doi: 10.1016/j.jenvman.2019.109872 31785457

[B68] Wang Z. Zhang H. Liu L. Li S. Xie J. Xue X.. (2022). Screening of phosphate solubilizing bacteria and their abilities of phosphorus solubilization and wheat growth promotion. BMC Microbiol. 22 (1), 296. doi: 10.1186/s12866-022-02715-7 36494624 PMC9733106

[B69] Wu Y. Cai P. Jing X. Niu X. Ji D. Ashry N. M.. (2019). Soil biofilm formation enhances microbial community diversity and metabolic activity. Environ. Int. 132, 105116. doi: 10.1016/j.envint.2019.105116 31479959

[B70] Xiao C. Chi R. Huang X. Zhang W. Qiu G. Wang D. (2008). Optimization for rock phosphate solubilization by phosphate-solubilizing fungi isolated from phosphates mines. Ecol. Eng. 33 (2), 187–193. doi: 10.1016/j.ecoleng.2008.04.001

[B71] Yagi R. Quadros T. C. F. Martins B. H. Andrade D. S. (2020). Maize yields and carbon pools in response to poultry litter, rock phosphate and p-solubilizing microorganisms. Sci. Agric. 77. doi: 10.1590/1678-992x-2018-0141

[B72] Yu Z. Duan X. Luo L. Dai S. Ding Z. Xia G. (2020). How plant hormones mediate salt stress responses. Trends Plant Sci. 25 (11), 1117–1130. doi: 10.1016/j.tplants.2020.06.008 32675014

[B73] Yu H. Wu X. Zhang G. Zhou F. Harvey P. R. Wang L. (2022). Identification of the phosphorus solubilizing bacteria strain JP233 and its effects on soil phosphorus leaching loss and crop growth. Front. Microbiol. 13. doi: 10.3389/fmicb.2022.892533 PMC9100411 35572684

