# Supplementary material for: Improving the efficiency of phosphate rocks combined with phosphate solubilizing Actinomycetota to increase wheat growth under alkaline and acidic soils
Source: Front Plant Sci. 2023 May 10;14:1154372. doi: 10.3389/fpls.2023.1154372 (PMC10206120; doi:10.3389/fpls.2023.1154372)
Supplement: Supplementary file 1 [file Table_1.docx]

Supplementary Material

**Improving the efficiency of phosphate rocks combined with phosphate solubilizing Actinomycetota to increase wheat growth under alkaline and acidic soils**

Kenza Boubekri^1,2^, Abdoulaye Soumare^1,3^, Karim Lyamlouli^1,2^, Yedir Ouhdouch^1,2^, Mohamed Hafidi^1,2*^, Lamfeddal Kouisni^4*^

*** Correspondence:** Corresponding Authors: [hafidi@uca.ac.ma](mailto:hafidi@uca.ac.ma) / [lamfeddal.kouisni@um6p.ma](mailto:lamfeddal.kouisni@um6p.ma)

# Supplementary Tables

**TABLE 1** │P-values of the MANOVA analysis at α= 0.001 between the different interactions evaluated under greenhouse conditions.

| Effect | Variables | F model | P value |
| --- | --- | --- | --- |
| *Soil* | **SDW** | 23.596 | 0.000*** |
|  | **RDW** | 36.483 | 0.000*** |
|  | **Spike** | 0.212 | 0.646^ns^ |
|  | **P content** | 1.582 | 0.211^ns^ |
|  | **K content** | 1.831 | 0.179^ns^ |
| *RP * Bacteria* | **SDW** | 19.496 | 0.000*** |
|  | **RDW** | 17.062 | 0.000*** |
|  | **Spike** | 24.858 | 0.000*** |
|  | **P content** | 11.381 | 0.000*** |
|  | **K content** | 5.788 | 0.000*** |
| *Soil * Bacteria * RP* | **SDW** | 5.762 | 0.000*** |
|  | **RDW** | 3.413 | 0.000*** |
|  | **Spike** | 9.919 | 0.000*** |
|  | **P content** | 13.743 | 0.000*** |
|  | **K content** | 6.994 | 0.000*** |

*** Significant at P <0.001; ns = non-significant


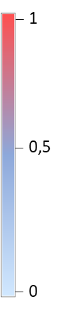

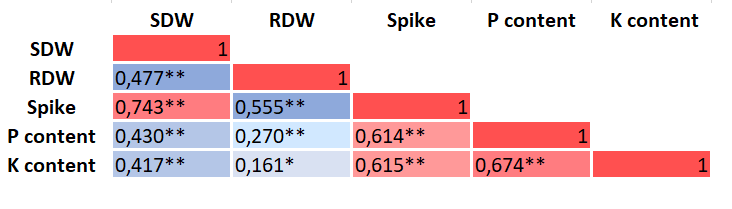


TABLE 2 │Pearson correlation matrix between the different agronomic parameters (n=5). * Significant at P <0,05, ** Significant at P< 0,01.

**
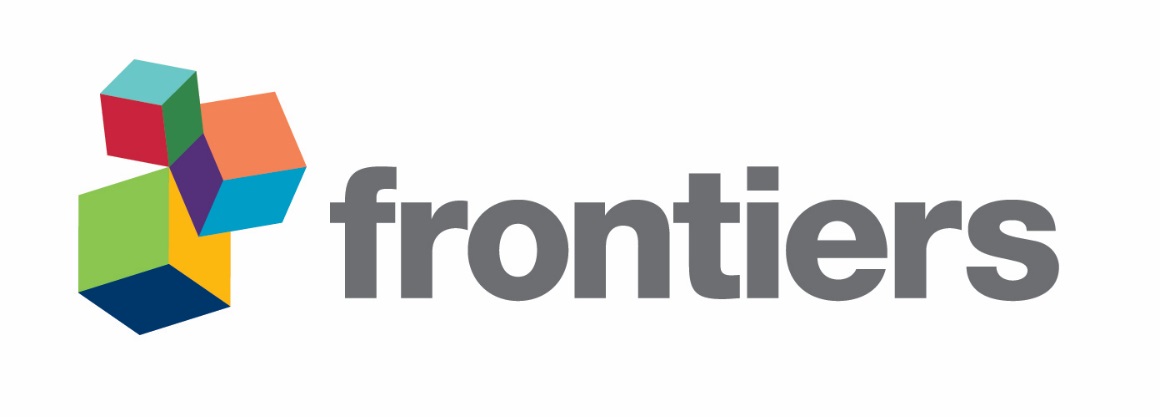
**
