# Supplementary figures and images for: Improving the efficiency of phosphate rocks combined with phosphate solubilizing Actinomycetota to increase wheat growth under alkaline and acidic soils
Source: Front Plant Sci. 2023 May 10;14:1154372. doi: 10.3389/fpls.2023.1154372 (PMC10206120; doi:10.3389/fpls.2023.1154372)

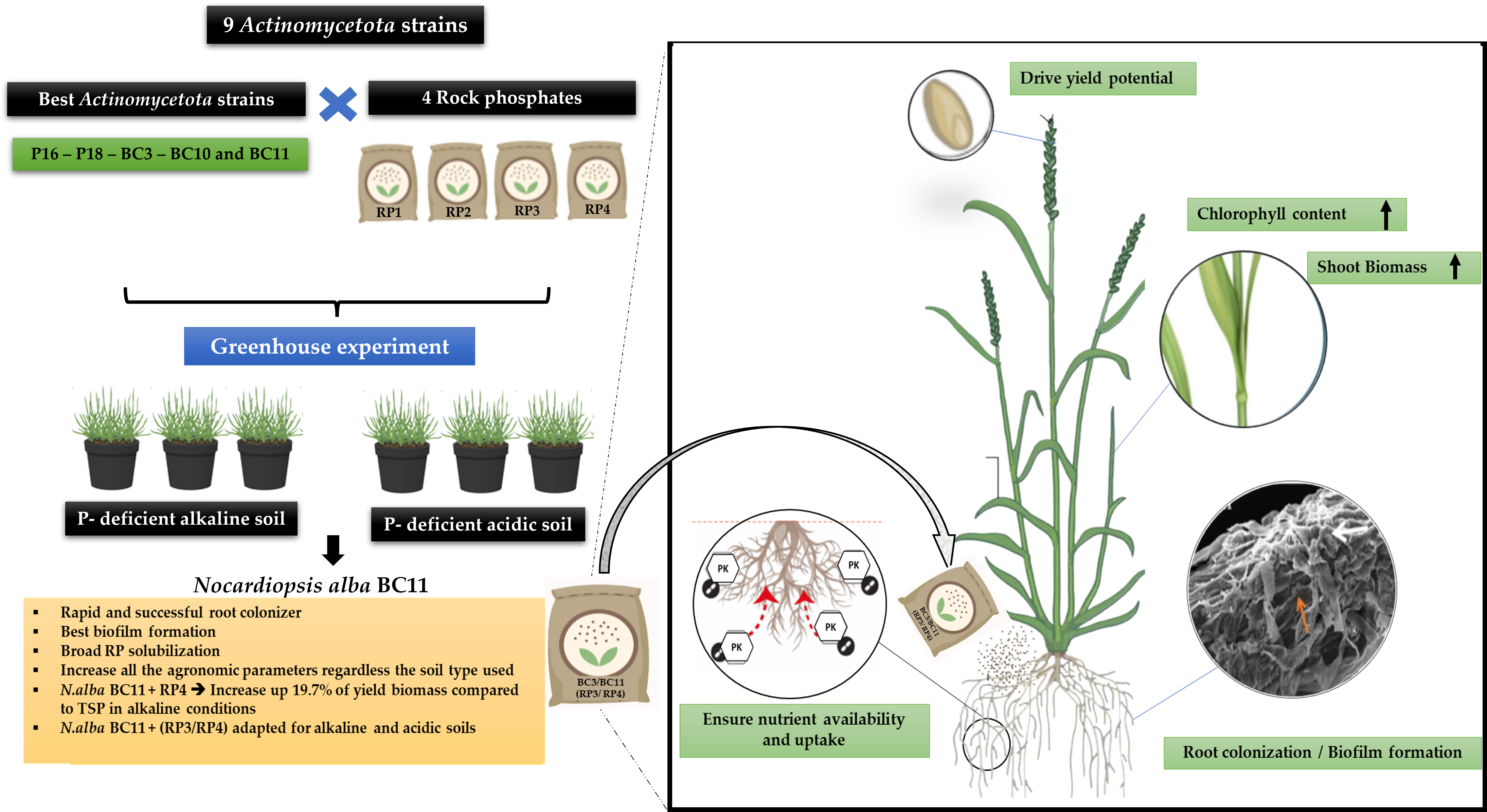

Supplement: Supplementary file 2 [file Image_1.tif]
